# Supplementary figures and images for: Mapping metabolic oscillations during cell cycle progression
Source: Cell Cycle. 2020 Oct 4;19(20):2676–84. doi: 10.1080/15384101.2020.1825203 (PMC7644150; doi:10.1080/15384101.2020.1825203)

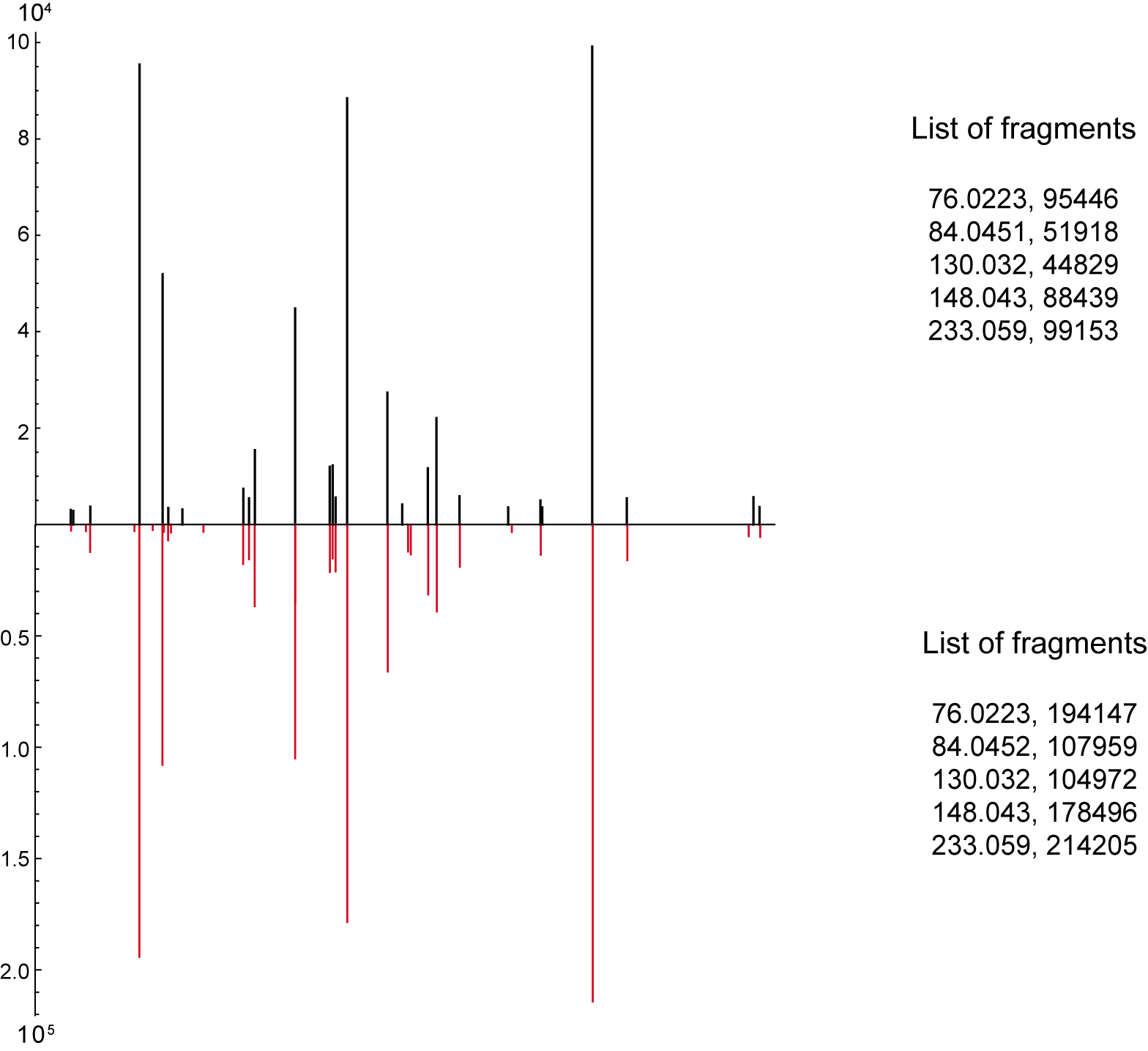

Supplement: Supplemental Material [file KCCY_A_1825203_SM3379.zip › Supplementary information/Supplementary Figure 1.tif]
